# Cluster randomized adaptive implementation trial comparing a standard versus enhanced implementation intervention to improve uptake of an effective re-engagement program for patients with serious mental illness

**DOI:** 10.1186/1748-5908-8-136

**Published:** 2013-11-20

**Authors:** Amy M Kilbourne, Kristen M Abraham, David E Goodrich, Nicholas W Bowersox, Daniel Almirall, Zongshan Lai, Kristina M Nord

**Affiliations:** 1 VA Center for Clinical Management Research, VA Ann Arbor Healthcare System, 2215 Fuller Road, Mailstop 152, Ann Arbor, MI 48105, USA; 2 Department of Psychiatry, University of Michigan Medical School, North Campus Research Complex, 2800 Plymouth Road, Building 16, Ann Arbor, MI 48109, USA; 3 Institute for Social Research, University of Michigan, 426 Thompson Street 2321, Ann Arbor, MI 48104, USA

**Keywords:** Adaptive designs, Mental disorders, Comparative effectiveness, Care management, Population health

## Abstract

**Background:**

Persons with serious mental illness (SMI) are disproportionately burdened by premature mortality. This disparity is exacerbated by poor continuity of care with the health system. The Veterans Health Administration (VA) developed Re-Engage, an effective population-based outreach program to identify veterans with SMI lost to care and to reconnect them with VA services. However, such programs often encounter barriers getting implemented into routine care. Adaptive designs are needed when the implementation intervention requires augmentation within sites that do not initially respond to an initial implementation intervention. This protocol describes the methods used in an adaptive implementation design study that aims to compare the effectiveness of a standard implementation strategy (Replicating Effective Programs, or REP) with REP enhanced with External Facilitation (enhanced REP) to promote the uptake of Re-Engage.

**Methods/Design:**

This study employs a four-phase, two-arm, longitudinal, clustered randomized trial design. VA sites (n = 158) across the United States with a designated Re-Engage provider, at least one Veteran with SMI lost to care, and who received standard REP during a six-month run-in phase. Subsequently, 88 sites with inadequate uptake were stratified at the cluster level by geographic region (n = 4) and VA regional service network (n = 20) and randomized to REP (n = 49) vs. enhanced REP (n = 39) in phase two. The primary outcome was the percentage of veterans on each facility outreach list documented on an electronic web registry. The intervention was at the site and network level and consisted of standard REP versus REP enhanced by external phone facilitation consults. At 12 months, enhanced REP sites returned to standard REP and 36 sites with inadequate participation received enhanced REP for six months in phase three. Secondary implementation outcomes included the percentage of veterans contacted directly by site providers and the percentage re-engaged in VA health services.

**Discussion:**

Adaptive implementation designs consisting of a sequence of decision rules that are tailored based on a site’s uptake of an effective program may produce more relevant, rapid, and generalizable results by more quickly validating or rejecting new implementation strategies, thus enhancing the efficiency and sustainability of implementation research and potentially leading to the rollout of more cost-efficient implementation strategies.

**Trial registration:**

Current Controlled Trials ISRCTN21059161.

## Background

Persons with serious mental illnesses (SMI), *e.g.*, bipolar disorder or schizophrenia, experience a disproportionate burden in morbidity and premature mortality from common medical conditions including cardiovascular diseases and certain cancers [1-3]. These physical health disparities may be exacerbated by long gaps in care from the healthcare system due to psychiatric symptoms or access barriers such as lack of transportation, insurance, or relationship with a primary care provider [4,5]. Many evidence-based practices help to mitigate these risks when this population remains engaged in care [6].

Continuity and coordination of care for vulnerable health populations with chronic conditions such as SMI are key components of the Chronic Care Model [7,8]. The Chronic Care Model is a population- and measurement-based approach that calls for healthcare organizations to use electronic registries to monitor vulnerable populations and to adjust treatment according to patient response. Not only has this model of care been successful in managing mental health across various healthcare settings [9,10], a number of large healthcare providers including the Veterans Health Administration (VA) have demonstrated that this model of care is effective for re-engaging persons with SMI who had been lost to care to prevent adverse health effects [11-13].

Despite the promise of the Chronic Care Model and similar population management programs, they are rarely routinely implemented in practice [14,15]. Several reasons that contribute to this research-to-practice gap have been described elsewhere [16] and include system and provider-level barriers to program uptake.

Identifying effective implementation interventions that address system and provider barriers can speed program uptake in routine practice. Implementation interventions are operationalized techniques based on an underlying framework or theory that are designed to enhance the uptake of effective programs across different healthcare settings [17]. Implementation interventions address multilevel barriers to program adoption, such as organizational culture, leadership buy-in, and provider training and capacity [16,18-24] to ultimately enhance program uptake [25].

Studies involving implementation intervention strategies have been referred to as type III hybrid-effectiveness implementation studies [17], where the intervention is the implementation strategy and the primary outcomes are focused on program uptake rather than testing the effectiveness of the program itself on patient outcomes.

A handful of type III hybrid effectiveness-implementation studies based on underlying implementation frameworks [26-30] have been recently conducted [17,31-34]. These studies involved highly specified implementation intervention strategies such as Replicating Effective Programs, Facilitation, or Evidence-based Quality Improvement that address multiple organizational and provider barriers [17,32,34]. These studies typically randomized sites to receive a new implementation strategy or standard dissemination to enhance the uptake of an effective program. Most of these studies take place in highly organized sites or treatment settings such as the VA.

Applying traditional randomized trial designs to complex implementation interventions can be challenging because they require several sites to achieve adequate power, and involve monitoring of both program and implementation intervention fidelity. Hence, these designs may not accommodate lower resourced sites that are less willing to be randomized or participate in study assessments [35]—the very sites implementation interventions are designed to assist. Moreover, not all sites may require the same level of implementation intervention, and some may require additional assistance due to underlying barriers to program adoption that are not apparent or measurable at baseline. This can lead to less cost-efficient use of implementation resources such as provider training, technical assistance, and the time require to build relationships with leaders and frontline providers across sites. In many situations, it is also unclear how long an implementation intervention is needed to improve program uptake [36,37].

In response to these challenges, we describe a new approach to implementation interventions. Increasingly used in clinical research, adaptive interventions guide the decisions to augment (change or adapt) existing interventions given signs of non-response (or other intermediate outcomes) during treatment [38-41]. When applied to implementation intervention studies, adaptive interventions allow sites that are not responding to an initial implementation strategy to receive an augmented implementation intervention. In contrast to simply measuring correlates of implementation non-response across sites, studies of adaptive implementation interventions can help to determine the added value of a more intensive implementation intervention strategy and how long the more intensive implementation strategy should be continued to achieve improved program uptake at individual sites.

### Aims and objectives

The aim of this study is to use an adaptive implementation design to compare the effectiveness of an enhanced versus standard version of an implementation strategy (Replicating Effective Programs, or REP) to promote the uptake of a population management program for patients with serious mental illness who have dropped out of care (‘Re-Engage’). REP is a previously operationalized implementation strategy that has been shown to improve the uptake of effective Chronic Care Model and related programs [28,31,42] and consists of program manual dissemination, training, and brief technical assistance. Enhanced REP includes standard REP with facilitation, which involves proactive coaching by a program expert that is focused on enhancing provider buy-in and uptake.

The primary implementation outcome is the uptake of the Re-Engage program, defined as the percentage of veterans’ with an updated documentation of their clinical status within 12 months, which is a central component of Re-Engage population management. The primary hypothesis is that among facilities not initially responding to standard REP, the addition of facilitation (enhanced REP) will be associated with increased percentages of documented updates to veterans’ clinical status. Secondary outcomes include facilities’ percentage of veterans who were provided brief care management, defined as percentage contacted or percentage returning to VA care. Additionally, we seek to explore whether among facilities that initially did not respond to standard REP the immediate addition of facilitation (enhanced REP) is associated with better outcomes than receiving Facilitation after a six-month delay.

## Methods

Described previously [5,11,13,43-48] Re-Engage is a VA nationally mandated brief care management program for veterans with serious mental illness [48]. At the time of protocol submission, the trial intervention had already started and collection of outcomes had begun. This study was reviewed and approved by the local VA Institutional Review Boards and was registered as a clinical trial (Current Controlled Trials ISRCTN21059161).

### Trial design

**Figure 1:**
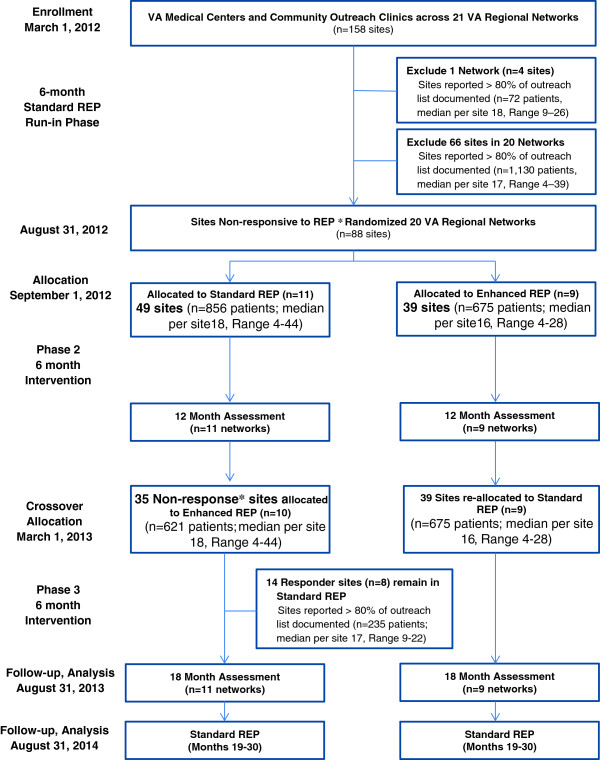
**Consort flow diagram for cluster randomized controlled trial.** (Footnote) *Non Response was defined using the as having less than 80% of patients on the site’s list with an updated documentation of clinical status in the web-based registry. Site response was defined as having ≥80% of patients on the site list with an updated documented clinical status.

**Figure 2:**
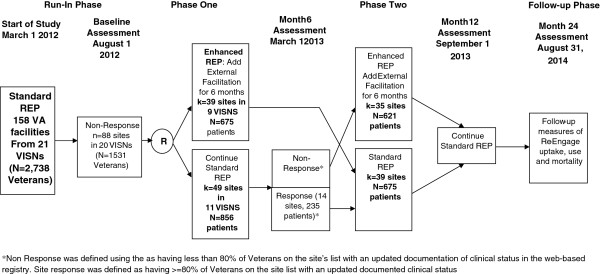
Trial design of continued standard REP versus enhanced REP (REP + external facilitation) among VA facilities non-responsive to standard REP.

This study employed a four-phase, two-arm, longitudinal, clustered randomized trial design (see CONSORT diagram- Figure 1). The four phases, described below, are: run-in, phase one, phase two, and follow-up (Figure 2). The unit of intervention in this study is the site. All VA sites in the 50 United States (N = 158) within the VA’s 21 regional networks that were required as part of the VA National Directive [48] to implement Re-Engage and had at least one veteran with serious mental illness who had dropped out of care were included and received standard REP in the run-in phase of the trial.

The six-month run-in phase included standard REP components, and at the end of the run-in phase, sites not responding to standard REP were identified. Phase one involved randomization to two site-level implementation interventions: Enhanced REP (adding Facilitation) or continuation of standard REP technical assistance on an as-needed basis. Phase two involved offering sites who were randomized to standard REP in phase one the enhanced REP facilitation, and discontinuing facilitation among sites who received facilitation in phase one.

### Run-in phase

During the initial run-in phase (1 March 2012 to 31 August 2012), all eligible VA sites nationally received standard REP to implement the Re-Engage program. As in the original study [11], providers implementing Re-Engage were asked to identify and document their patients' current disposition based on a pre-generated list of those who had dropped out of care, as well to attempt to contact them and invite them back to VA health services. The initial run-in phase began when the requirement to implement the program was communicated to sites in March 2012 and continued until 31 August 2012 [48]. During this phase, the designated mental health provider was identified at each eligible facility, and he or she received a computerized list of patients who had dropped out of care and a website link to track their status, a package describing the Re-Engage program, training, and brief technical assistance.

### Phase one

Sites with inadequate implementation of Re-Engage (*i.e.*, non-responding sites) as of 1 September 2012 were then identified based on a previously established eligibility criterion and randomized to receive enhanced REP or continued REP. Inadequate implementation of Re-Engage was defined as documenting and attempting to contact less than 80% of patients on the drop-out list, based on a review of the website registry from each site. This previously established measure is considered a core component of the Re-Engage program because it is an indicator of whether the provider reviewed the list and attempted to find the patient. This measure was used to benchmark implementation response because this measure is most likely to be impacted by individual providers. A cut-point of 80% was selected because it is a standard definition used to determine adequate adherence to practice guidelines based on the Agency for Healthcare Research and Quality [49].

Sites having successfully implemented Re-Engage by the end of the run-in phase (31 August 2012) continued to receive standard REP for phases one, two, and the follow-up phase of the trial (Figure 2).

Sites that had not adequately implemented Re-Engage as of the end of the run-in phase (*i.e.*, non-responding sites) were stratified by geographic region and 1:1 randomized by the sites’ VA regional network (n = 20) to enhanced or continued standard REP. Because each VA regional network has mental health leaders that communicate across sites, randomization was conducted at the VA regional network level to minimize potential for contamination. Non-responding sites randomized to receive enhanced REP received six months of facilitation and those randomized to standard REP received technical assistance calls only if they requested it for six months during phase one.

### Phase two

After the end of phase one (28 February 2013), sites originally randomized to receive standard REP and who were still non-responsive (<80% documentation of patients’ clinical status) received enhanced REP facilitation. Sites that were initially randomized to receive standard REP and met the implementation benchmark as of the end of phase one continued to receive standard REP during phase two. Sites receiving enhanced REP during phase one received standard REP in phase two regardless of responsiveness.

### Follow-up phase

After the end of phase two of the study (31 August, 2013), standard REP will continue and outcomes including VA use [50] will be monitored through 31 August 2014 using previously established methods [51,52].

### Participants

This implementation trial is being conducted at the VA facility-level between January 2012 and August 2013. A VA facility was eligible for the current trial if it was included in the national VA Re-Engage program. VA facilities were included in the national Re-Engage program if they were within the 50 United States or Puerto Rico, were required, per VA policy [53], to have a mental health provider who filled the role of a Local Recovery Coordinator, and had at least one veteran with serious mental illness who was lost to care—*i.e.*, had been seen at the facility in fiscal year (FY) 2008 or FY 2009, but had no subsequent outpatient visits or an inpatient stay of less than two days as of January 2012. There were a total of 158 facilities eligible for Re-Engage, of which 139 were medical centers (*i.e.*, with hospital beds) and 19 were community-based outpatient clinics.

### Setting and target population

Re-Engage is a national VA program which has three core components: panel management, brief care management, and proactive outreach services that are designed to re-engage in VA healthcare veterans with serious mental illness (*i.e.*, schizophrenia or bipolar disorder) who previously received VA healthcare, but have not been seen in VA healthcare for at least one year. Re-Engage was initially developed by VA Office of Medical Inspector as a quality improvement program based on awareness that veterans with serious mental illness face high rates of medical comorbidities that require regular medical care [43-45], and that gaps in healthcare services among this population contribute to early mortality [5,46]. The VA Office of Medical Inspector quality improvement program was completed in 2010 and found that veterans with SMI who returned to care had lower rates of mortality (0.3%) than veterans who were targeted for re-engagement, but did not return to care (3.9%) [11,47]. As a result, VA mandated that Re-Engage be implemented as part of standard clinical care [48].

One provider at each VA facility, specifically the Local Recovery Coordinator, [53], is designated to implement Re-Engage components at his or her facility. Local Recovery Coordinators are typically social workers or psychologists who have both administrative and clinical duties [53]. Re-Engage core components were designed to be part of Local Recovery Coordinator’s clinical duties [48], and include the following: panel management: receiving a list of veterans with SMI whose last VA healthcare visit was at their facility, reviewing the medical record and other informational sources to locate the veterans, updating their clinical status or disposition (*e.g.*, vital status, whereabouts, etc.) in a web-based clinical registry; outreach*: i.e.*, attempting to contact the veterans in person, via telephone, or mail; and brief care management: completing a semi-structured assessment of veterans current health status and healthcare needs and inviting veterans to return to VA care and assisting with the scheduling of any desired VA appointments.

As part of the Re-Engage program, the VA National Serious Mental Illness Treatment Resource and Evaluation Center (SMITREC) provides the Local Recovery Coordinator at each facility with lists containing the names, last known contact information, and last known recent clinical history for the veterans for the Re-Engage program. Based on previously described processes [11,13], patients were eligible for Re-Engage if they had at least one diagnosis of schizophrenia (International Classification of Diseases, Ninth Revision, Clinical Modification (ICD-9-CM) codes 295.0–295.4; 295.6–295.9) or bipolar disorder (ICD-9-CM codes 296.0–296.8) recorded in an inpatient or outpatient visit in FY 2008 or FY 2009; had not been seen in VA care for at least one year (*i.e.*, dropped out of care: defined as no recorded outpatient visits or an inpatient stay of less than two days in the FYs after their last year with any visit); and were still alive as of March 2012 based on currently available mortality information from the VA Beneficiary Identification and Records Locater Subsystem (BIRLS), a well-established resource for VA mortality data, the Social Security Administration Death Master File, and the National Death Index [54].

As of March 2012, a total of 5,240 veterans were identified through Re-Engage. The average number of identified veterans at each facility was 33.2 (standard dev = 22.5), with a range of 4 to 147. Of these, a subset of veterans were targeted as high-priority for the Re-Engage program if they had at a history of at least one inpatient hospitalization prior to drop out and were less than 65 years of age (*i.e.*, less likely to be in a nursing home or covered by Medicare services). All high priority veterans were included on initial lists that were disseminated to facilities in March 2012. Additional veterans (up to a total of 42 per facility) were included on initial lists based on the dates they were last seen in VA healthcare. Each facility’s initial list contained no more than 42 veterans in order to provide Local Recovery Coordinators with a manageable number of veterans to contact. In March 2012, contact information for a total of 2,733 veterans was disseminated to facilities on the initial lists (n = 2,733, mean per facility = 17.3, std dev 6.3, range: 4 – 42). Contact information for the remaining 2,507 veterans (‘second list’) identified in March 2012 was disseminated in July 2012.

Although Re-Engage is an ongoing clinical program and over time additional veterans who have been lost-to-care will continue to be identified and their names disseminated to local Recovery Coordinators at each facility, this implementation study focused on the first list of veterans (n = 2,733) identified and disseminated in March 2012, and outcomes will be measured on the basis of this cohort.

### Randomization

In phase two of the trial, facilities with insufficient implementation of Re-Engage were stratified by geographic region and randomized at the VA integrated services network-level to receive enhanced REP or continue receiving standard REP. We stratified by geographic region because preliminary analyses indicated that uptake of Re-Engage at the end of phase one differed by geographic region. Randomization was conducted by the study program analyst and occurred at the veterans integrated service network-level as opposed to the facility level because in enhanced REP External facilitation involved communications with regional VA leadership and we sought to minimize the potential for contamination across facilities within the same integrated service network. Because the providers involved in the implementation knew that they were receiving enhanced versus standard REP, allocation concealment and blinded randomization were not applicable.

### Implementation interventions

REP is based on the Centers for Disease Control and Prevention’s Research-to-Practice Framework [28,29,42]. Derived from Social Learning Theory [55] and Rogers’ diffusion model [56], REP consists of three central operational components: program ‘packaging’ (*i.e.*, translation of treatment materials into user-friendly language), provider training, and brief technical assistance for providers to address barriers to uptake. The combination of these three components compared to package dissemination alone resulted in improved uptake and fidelity to HIV prevention intervention programs in AIDS service organizations [42,57].

Although standard REP employs key tactical strategies that can promote effective adoption of effective programs [42,58], it was not designed address multilevel barriers to implementation, such as competing demands on providers and limited leadership support for new programs [59]. Hence, REP was enhanced by including facilitation based on the Promoting Action on Research Implementation in Health Services (PARiHS) framework [58,60-62]. Facilitation is a systematic and iterative process in which implementation experts promote program uptake by working with frontline providers to identify and mitigate barriers to program adoption [63,64].

Enhanced and standard REP implementation intervention components [11,13] that were developed for this current study are described previously [13]. In brief, standard REP consists of dissemination of a Re-Engage package describing the program’s core components (*e.g.*, registry of patients lost to care, website to document patient status), training the mental health providers implementing Re-Engage (*e.g.*, using the registry and website, contacting patients, and routing them to services), and brief technical assistance calls to the mental health providers who were to implement Re-Engage (Table 1). Enhanced REP included additional External Facilitators [65] with backgrounds in psychology who made calls on a weekly basis to mental health providers to provide specific guidance to overcoming barriers to implementing Re-Engage (Table 1). Facilitators also reached out to VA regional mental health leaders to promote the program and provided feedback on implementation progress based on monthly reports on uptake across sites.

**Table 1:** Implementation components of standard REP and enhanced REP

**Component**	**Description**	**Standard REP**	**Enhanced REP**
Package	Implementation guide was disseminated to all providers at eligible sites, describing the Re-Engage program, a list of frequently asked questions, sample documents for program tasks, program policies, data security, and related research.	**√**	**√**
Training	Three 1.5-hour national conference call trainings of mental health providers on how to conduct program. Program materials made available on mental health provider website. Research staff available to answer questions via email or telephone.	**√**	**√**
Technical Assistance	Ongoing assistance via 1-hour biweekly conference calls led by study staff for mental health providers to answer technical questions on Re-Engage program implementation and study staff were available on an ad-hoc basis to answer questions from individual providers. Monthly reports were generated to track Re-Engage uptake (% patients with updated clinical status documented).	**√**	**√**
	Sites receiving standard REP technical assistance in phases one and two did not receive calls but study staff were available if they were contacted on an as –needed basis to address technical questions regarding Re-Engage implementation.		
**External Facilitation**			
Gather information	Facilitators gather information from various sources (monthly evaluation reports, VISN Mental Health Leadership, mental health providers, VA Mental Health Services Leadership) to identify potential facility-specific barriers and facilitators to implementation.		**√**
Ongoing partnership support	Weekly phone calls with Facilitators, Technical Assistance staff, and VA national leaders involved in national Re-Engage program and VA mental health services. Facilitators maintain open communication with VA leaders regarding implementation nationally and at specific sites through these phone calls and email communication. Facilitators also maintain ongoing contact with one another through separate weekly meetings.		**√**
Garner regional and local support	Facilitators initiate contact with regional mental health leadership affiliated with local sites, providing information regarding Re-Engage program implementation and added value. Maintain ongoing contact and request support from regional leadership as indicated.		**√**
Identify barriers and facilitators	Facilitators and mental health providers hold monthly calls for six months and collaboratively identify each facility’s specific challenges (*e.g.*, time, resources) to program implementation as well as potential assets (*e.g.*, consistency with other initiatives, support from local leadership) to program implementation.		**√**
Collaboratively develop action plans	Facilitators assist mental health providers in identifying what specific actions they can take to implement program.		**√**
Feedback and Link to available resources	Facilitators provide feedback to mental health providers regarding implementation and action plan progress. Facilitators refer mental health providers to existing resources, including the Technical Assistance available through standard REP, existing documents regarding the program intervention, facility-level, regional, or national leadership.		**√**

### Outcomes

#### Primary implementation outcome

The primary measure of the uptake of Re-Engage was the percentage of veterans on each facility’s list whose clinical status was updated in the Re-Engage web-based clinical registry. The Veteran Clinical Status Updated measure was calculated as the number of veterans with an update on their clinical status or disposition in the web-based registry compared to the total number of veterans on each facility’s list. This outcome measure indicates whether facilities are actively attempting to locate and contact the veterans who have dropped out of care and is independent of whether the veterans are reachable, and thus is a good indicator of implementation. Consistent with recommendations from previous studies of cut-points used to establish adequate adherence to clinical processes or guidelines [66,67], inadequate uptake of Re-Engage is defined as whether an updated clinical status was available for less than 80% of the veterans on a given facility list. Although this measure is tabulated monthly for the purposes of the VA Re-Engage program, for the purposes of this implementation study, this outcome measure was examined at the end of the run-in period (end of August 2012), the end of phase one (end of February 2013), the end of phase two (end of August 2013), and during the follow-up phase (end of February 2014, end of August 2014).

#### Secondary outcomes

Secondary measures of implementation include the percentage of veterans that the Local Recovery Coordinators successfully contacted among those who were on their lists and still alive and able to be contacted (*e.g.*, had available address or phone number, no documentation of institutionalization or incarceration), and the percentage of veterans contacted who re-engaged in VA healthcare services. These measures, percentage of veterans contacted and percentage of veterans Re-Engaged, reflect the brief care management strategies that are part of Re-Engage.

Additional secondary outcome measures include veteran-level variables. All-cause mortality and utilization of VA healthcare services will be compared as a function of whether or not veterans were able to be contacted, and if contacted, whether or not they indicated an interested in returning to VA healthcare. Healthcare utilization variables will include number and length of stay of inpatient medical and mental health hospitalizations, number of outpatient mental health and general medical visits, and number of emergency department visits. Utilization of mental healthcare will be further examined by identifying the number of visits to recovery-oriented mental health services (*e.g.*, psychosocial rehabilitation and recovery centers, supported employment services), as these services are targeted to veterans with SMI [53]. Moreover, because a large proportion of homeless veterans have a psychiatric diagnosis [51,52], the number of visits to VA homeless program services will be examined.

### Analysis

To examine the implementation of Re-Engage, we will use generalized estimating equations repeated measures models to examine facility-level changes in the percentage of veterans with an updated clinical status, percentage of veterans contacted, and percentage of veterans re-engaged over time (end of phase one, end of phase two, end of phase three, follow-up periods), controlling for veteran-level (*i.e.*, percentage of veterans on a facility’s list with particular demographic or clinical characteristics) and facility-level characteristics. The covariates section below and Table 2 contain specific veteran-level and facility-level variables.

**Table 2:** Potential covariates of re-engage implementation

**Covariates for all facilities**	**Data source**	**Construct from CFIR**
Patient characteristics:	Administrative Data- NPR	
• Gender		
• Race		
• Age		
• Military service period		
• Psychiatric Diagnoses		
• Indication of a history of homelessness		
• History of substance use disorder		
Presence of PRRC at site	Administrative Data- MHS maintained	Inner Setting- Implementation Climate- Compatibility
Presence of LRC when Re-Engage was Rolled Out	Administrative Data- MHS maintained	Inner Setting- Readiness for Implementation- Available Resources
Facility Complexity	Administrative Data	Inner Setting- Structural Characteristics
Number of Vets on List	Re-Engage Program Records	Intervention Characteristics- Complexity
Overall facility size	Administrative Data	Inner Setting- Structural Characteristics
Number of SMI Vets associated with Facility	Administrative Data- NPR	Inner Setting- Structural Characteristics
Academic Affiliation of facility	Administrative Data- Ascertained via US News & World Report Med School Rankings	Inner Setting- Culture
Urban/Rural Facility	Administrative Data	Inner Setting- Structural Characteristics
Whether the site or VISN viewed Re-Engage as a research project	Technical Assistance Minutes	Either: Characteristics of Individuals- Knowledge & Beliefs about the Intervention OR Characteristics of the Intervention- Intervention Source
The performance of other VAs in VISN on SMI Re-Engage Implementation	Re-Engage Program Records	Outer Setting- Peer Pressure
**Covariates for sites receiving enhanced REP**	**Data source**	**Construct from CFIR**
Number of Facilitation Contacts with Site	Facilitator Notes	Process- Executing
Number of Facilitation Contacts with VISN Mental Health Leadership	Facilitator Notes	Process- Executing
Did the Facility adapt SMI Re-Engage and use a Team approach?	Facilitator Notes	Intervention Characteristics- Adaptability
Was there evidence that SMI Re-Engage was a priority in VISN or at site?	Facilitator Notes	Inner Setting- Implementation Culture- Relative Priority
Did the LRC perceive available time (or resources) in order to do the required aspects of the SMI Re-Engage program?	Facilitator Notes	Inner Setting- Readiness for Implementation- Available Resources
Does the VISN Mental Health Lead/seem to have a positive view of SMI Re-Engage?	Facilitator Notes	Characteristics of Individuals- Knowledge & Beliefs about the Intervention
Does the LRC at a site seem to have a positive view of SMI Re-Engage?	Facilitator Notes	Characteristics of Individuals- Knowledge & Beliefs about the Intervention
Does the VISN Mental Health Lead accurately understand SMI Re-engage?	Facilitator Notes	Characteristics of Individuals- Knowledge & Beliefs about the Intervention
Does the LRC accurately understand SMI Re-Engage?	Facilitator Notes	Characteristics of Individuals- Knowledge & Beliefs about the Intervention
Does the LRC feel capable of executing the tasks associated with SMI Re-Engage?	Facilitator Notes	Characteristics of Individuals- Self-efficacy

**Abbreviations**: *NPR* the VHA National Psychosis Registry, *CFIR* the Consolidated Framework for Implementation Research, *PRRC* VA Psychosocial Rehabilitation and Recovery Center, *MHS* VHA Mental Health Services, *VISN* VA Veterans Integrated Service Network, *SMI* Serious mental illness, *LRC* Local recovery coordinator.

To examine the effects of contacting veterans and re-engaging veterans on patient-level outcomes of mortality and healthcare utilization, we will use generalized mixed effects models to account for VISN and facility-level covariates as well as patient-level characteristics. We will employ logistic regression models or Poisson models as appropriate, based on whether dependent measure is dichotomous (*e.g.*, mortality) or a count variable (*e.g.*, number of hospitalizations).

### Sample size

Our study included all eligible VA facilities (n = 158) within the 21 VA integrated service networks (VISNs) that had a provider to implement Re-Engage at the time of this study. All 158 VA facilities received standard REP in phase one of this trial. Among these, at the end of phase one, 88 facilities (55.7%) in 20 of the 21 VISNs had updated the clinical status of less than 80% of veterans’, indicating insufficient implementation of Re-Engage. These 20 VISNs (containing 88 facilities with insufficient implementation of Re-Engage) entered phase two of the trial and were thus randomized (as described above) to continue standard REP or receive enhanced REP. Through randomization, nine VISNs that included facilities were assigned to enhanced REP, and the remaining 11 VISNS that included 49 facilities were assigned to standard REP.

### Statistical power considerations

The data analysis plan for the primary aim is a two-sample comparison of facilities within VISNs randomized to enhanced versus standard REP. Based on the sample sizes described above, we conducted analyses to determine whether we had adequate statistical power to detect a significant difference in our primary (percentage of veterans with updated clinical status) and secondary (percentage of veterans contacted, percentage veterans Re-Engaged) facility-level implementation outcomes between the two groups of facilities.

At the end of phase one, the average percentage of veterans with and updated clinical status (primary implementation outcome) among the 88 underperforming facilities was 22% (SD = 25%). To account for the between-VISN variation induced by the within-VISN correlation in the average rate of the percentage of veterans with updated clinical status, we inflate the variance term in the standard sample size formula by 1 + (n-1)*ICC where ICC is the VISN interclass correlation coefficient for the average percentage of veterans with updated clinical status. The ICC for the average percentage of veterans with updated clinical status among the 20 VISNs entering phase two was 0.177. Using a two-sided, two-sample *t* test based on the sample sizes given above, a Type-I error rate of 5%, an ICC = 0.177, we will have 80% power to detect an effect size of 0.72 (Cohen’s D). This effect size corresponds to a between-site difference of 21 percentage points in the percentage of veterans with an updated clinical status.

For the secondary outcome the percentage of veterans contacted, based on the initial values of 35% (SD = 29%), and ICC = 0.31, with 80% power we will can detect an effect size of 0.78 (Cohen’s D), which corresponds to an approximate difference of 22 percentage points. Similarly, for the secondary outcome, the percentage of veterans re-engaged in care, based on the initial values of 26% (SD = 38%), we will have 80% to detect an effect size of 0.88 (Cohen’s D), or a difference of 33 percentage points.

### Covariates of implementation outcomes

Drawing on the Consolidated Framework for Implementation Science (CFIR) [68] and the PARiHS Framework, we identified organizational and facility as well as patient-level variables that we will adjust for in examining implementation of Re-Engage. Covariates are listed in Table 2, along with the constructs they represent based on CFIR, and the data source(s) that will be used to ascertain the variable.

Data sources for patient-level data will include information ascertained from national VA administrative databases (*e.g.*, National Psychosis Registry) as well as information gathered through Local Recovery Coordinators’ contacts with veterans. Data sources for organizational and contextual factors will include VA administrative records and the all-employee survey, minutes from standard REP technical assistance calls, and surveys completed by facility clinicians and administrators. Surveys were disseminated to facility Local Recovery Coordinators, Homelessness Coordinators, and Mental Health Service Line Leaders beginning in March 2012. The surveys sent to the Local Recovery Coordinators and Homeless Coordinators ascertained their job duties and the frequency of their interactions with clinicians in other roles. Mental Health Service Line Leaders were provided with the Mental Health Program Survey [69] to assess structural and organizational characteristics of mental health services at the facility. For facilities that received enhanced REP, Facilitators’ notes on contacts with facilities and stakeholders will serve as additional data sources.

Qualitative data from Facilitators’ notes and technical assistance minutes will be coded to identify possible barriers and facilitators to program implementation. Although many of the potential covariates were identified *a priori* (*e.g.*, structural and organizational characteristics), additional potential covariates have been identified through standard REP and enhanced REP processes.

### Monitoring implementation intervention

A central component of the Re-Engage adaptive implementation intervention design was documenting and monitoring activities related to the implementation interventions. A primary reason for documenting these activities was not only to monitor whether the implementation interventions are being conducted with fidelity, but to monitor the time and effort required in order to determine the added costs of enhanced REP over time. For sites that received enhanced REP, Facilitators completed a regular log form for each contact with each site provider, and notes from these contact sheets serve as additional data sources to monitor facilitation activities and time (Table 3). Data from Facilitators’ notes and minutes from the Facilitators’ weekly meetings will be coded using a previously established fidelity tool for enhanced REP [13] to determine to what extent Facilitators utilized core components of facilitation. The tool will assess core tasks of facilitation, notably whether the Facilitators identified possible barriers and solutions to Re-Engage implementation at each site, and whether site providers used Facilitator recommendations.

**Table 3:** Documentation of enhanced REP Facilitators’ Core Component Tasks and Time

**Core facilitation task**	**Implementation step**
Preparation for communication with facilities or regional leadership	• Review implementation progress
	• Review barriers or facilitators to implementation described during last contact or ascertained through other sources as documented in Facilitator database
	• Review stated actions planned from last contact (*i.e.*, action plans) as documented in Facilitator database
Semi-structured communication with facilities and/or regional stakeholders (phone call)	• Discuss progress on action plans that were established in prior contact
	• Discuss implementation progress based on monthly report
	• Provide support, encouragement, reinforcement of progress made
	• Collaboratively identify additional/existing barriers, changes to context that could affect implementation
	• Problem-solve strategies, solutions to address barriers
	• Collaboratively identify additional/existing facilitators and discuss how to use them to encourage implementation
	• Provide suggestions for how to adapt intervention to local setting without compromising core components
	• If needed, refer to technical assistance resources (available through standard REP)
	• Provide information in response to questions, concerns, or promise to obtain needed information
	• Collaboratively identify specific actions that can be taken to assist in implementation prior to next contact
Follow up	• Number of contacts with facility mental health provider implementing Re-Engage
	• Number of contacts with regional network leaders
	• Number of barriers and solutions discussed with facility providers
	• Follow-up emails and phone calls to link to existing resources (*e.g.*, technical assistance, leadership, continue problem solving a specific issue, provide information in response to a question
	• Schedule next contact (*e.g.*, schedule conference call lines, email facilities that have not been responsive)
Facilitators weekly communication with leadership partners (phone call)	• Facilitators join the weekly calls between research staff and VA national mental health leadership
	• Provide overview of facilitation progress
	• Provide information to/seek information from VA Mental Health Services Leadership about facility-specific issues that may have arisen during the week’s facilitation communications, seek guidance as needed
	• Obtain information regarding other initiatives affecting mental health providers
Facilitators weekly peer consultation meeting	• Review each facility receiving facilitation, identify implementation progress, barriers
	• Discuss strategies being used to encourage implementation at each facility
	• Provide support, encouragement, and accountability to one another
	• Provide information to/seek information from Technical Assistance research staff, as needed

## Trial status

To date the run-in phase and phase one of the implementation trial have been completed, and phase two of the trial is ongoing at this time. All 39 sites within 9 VA regional networks that received enhanced REP during phase one have now returned to receiving standard REP. Of the 49 sites within 11 VA regional networks that were randomized to standard REP during phase one of the trial, 36 sites (73.5%) in 10 VA regional networks continued to have less than 80% of their patients on their list with updated clinical status at the end of phase 1 and will receive enhanced REP.

## Discussion

This paper describes to our knowledge one of the first adaptive implementation intervention trial designs. The study is testing the effectiveness of facilitation as an adjunct to standard REP among non-responding sites on the implementation of a national VA program, Re-Engage, which is designed to assist patients with serious mental illness who have dropped out of care return to VA services.

An adaptive implementation design was optimal for this particular study because enhanced REP included additional personnel time and effort that may, over time, be too expensive to implement. Not all sites may need a more intensive implementation intervention (enhanced REP facilitation) to promote the uptake of an effective program. Hence, the adaptive nature of this trial randomized sites that required additional assistance, which allowed for more efficient use of facilitation resources. In addition, comparing the timing of added facilitation (immediately after observed non-response in phase one or six months later during phase two) provided an opportunity to focus on the impact of facilitation among later adopters of effective programs at the site level.

In addition, this study was also to date one of the first implementation intervention trials that took advantage of a population-based, national rollout of an effective program within a US health system. Hence, the study sought to use the VA’s national mandate to implement Re-Engage as the foundation for a natural experiment to test different implementation intervention strategies. The VA was an ideal setting in which to conduct this adaptive implementation trial because of the availability of national patient data and provider networks to identify those who had dropped out of care and to monitor subsequent use and outcomes at the patient and site levels. By including all sites that were required to implement Re-Engage per the mandate, there was opportunity to test the implementation interventions among sites that were less likely to initially respond to a lower-cost implementation strategy (REP). Previous implementation intervention trials often had to seek permission first from sites to participate in a study, often leading to potential cherry-picking and selection effects that may skew implementation results [19].

Another advantage in conducting an adaptive implementation strategy is that measuring site contextual factors that might influence program uptake is not required. Instead, sites are included in the randomization portion of the study only if they are not responding to an initial implementation strategy, and the reasons for non-response may not be observable or measurable even with organizational assessments. Moreover, because the national rollout of Re-Engage occurred relatively quickly, the window of opportunity to ascertain organizational data across all of the sites to predict implementation non-response was limited. Several organizational assessments exist [70], but to date most have not been systematically used to identify predictors of program uptake, and those predictors may vary depending on the particular program.

There are limitations to this type of design that warrant consideration, especially when deciding to replicate the design in other settings. Notably, adaptive implementation intervention designs are potentially less feasible in settings without large numbers of sites that are either willing or mandated to provide the effective program, and have access to common data sources to gauge patient outcomes. Second, the large number of sites precluded more intensive monitoring of program fidelity beyond documentation by the frontline provider via the website. Third, cost considerations precluded having Facilitators as part of the enhanced REP intervention make site visits or involve local site leaders on a more regular basis. At least one prior study or enhanced REP included an Internal as well as External Facilitator who can provider more on-the-ground coaching and guidance to the frontline mental health provider, linking them to leaders and resources not available or known to an outside External Facilitator [31-34].

## Conclusions

The results of this study will yield new information on how to conduct adaptive implementation intervention trials at the national level. These findings will have the potential to inform not only further implementation research, but also the actual implementation of effective programs in large healthcare settings. This study also sets the stage for determining the added value of more intensive implementation interventions within sites that need additional support to promote the uptake of effective programs. Ultimately, adaptive implementation designs may produce more relevant, rapid, and generalizable results by more quickly validating or rejecting new implementation strategies, thus enhancing the efficiency and sustainability of implementation research and potentially lead to the rollout of more cost-efficient implementation strategies.

## Competing interests

The authors have no conflicts of interest—financial or non-financial—in the methods described in this manuscript.

## Authors’ contributions

All authors have made substantial contributions to conception and design, or acquisition of data, or analysis and interpretation of data, have been involved in drafting the manuscript or revising it critically for important intellectual content, and have given final approval of the version to be published. AK conceptualized the study, acquired funding, developed the implementation framework and interventions, and drafted the manuscript. AK, DG, NB, KA, and KN contributed to the development of the implementation intervention tools and contributed to the editing of the manuscript; AK, DA, DG, and ZL contributed to the methods and design of the manuscript draft and final revisions. All authors read and approved the final manuscript.
